# Under ONIOM Layers: Analysis of BCR-ABL Enzyme Inhibitors Through Bond-Critical Points and Natural Orbitals

**DOI:** 10.3390/molecules30204145

**Published:** 2025-10-21

**Authors:** Kelvyn M. L. Rocha, Érica C. M. Nascimento, João B. L. Martins

**Affiliations:** 1 Department of Pharmacy, Faculty of Health Sciences, University of Brasilia, Brasilia 70910-900, DF, Brazil; kelvynmagalhaeslr@gmail.com; 2 Laboratory of Computational Chemistry, Institute of Chemistry, University of Brasilia, Brasilia 70910-900, DF, Brazil; ericacristinamoreno@gmail.com

**Keywords:** ONIOM, ponatinib, rebastinib, CML, NBO, BCP

## Abstract

Considering the relevance of hydrogen bonds and other intermolecular interactions in regulating the activity of the tyrosine kinase class of enzymes, an in-depth electronic structure study of these forces in the context of the BCR-ABL protein was performed through full optimizations using the ONIOM method. Rebastinib and ponatinib were docked to the target enzyme using AutoDock Vina to provide starting-point geometries, which were then optimized through ONIOM calculations. This study evaluated Frontier Molecular Orbitals (FMOs) and Bond-Critical Points (BCPs) located in the sites of interactions formed with accessible residues, such as Glu286, Met318, and Asp381. Ponatinib’s ONIOM-optimized structure was shown to not only form and preserve prominent interactions, which were shown to be significantly stronger than those formed by rebastinib, but also to be associated with a significant increase in the HOMO (Highest Occupied Molecular Orbital)−LUMO (Lowest Unoccupied Molecular Orbital) gap, indicating its potential to hinder catalytic activity by providing higher chemical stability when compared to rebastinib.

## 1. Introduction

The BCR-ABL transcript is part of the tyrosine kinase class of enzymes, being related to the development of Chronic Myeloid Leukemia (CML) and some forms of severe Acute Lymphoblastic Leukemia (ALL) [1,2,3,4,5,6,7,8,9]. This enzyme has its origin in a mutual translocation of genetic material involving the 9th and 22nd pairs of chromosomes, which produces a chimeric arrangement—the Philadelphia chromosome—that contains the mutant BCR-ABL gene [10,11,12,13]. In the context of CML, the detection of this gene through karyotyping or in situ hybridization tends to be among the best techniques for diagnosis [1,14,15,16].

The oncogenic activity begins with an essential phosphorylation of the Tyr177 residue, which promotes the recruitment of other cytoplasmic proteins to form a complex structure with multiple protein subunits [17]. The first of these proteins is the Growth Factor Receptor-Bound Protein 2 (GRB2), which joins BCR-ABL and results in a complex capable of recruiting the Son of Sevenless (SOS) factor [9]. The BCR-ABL/GRB2/SOS complex promotes activation of the Rat Sarcoma (Ras) factors through its conversion to the GTP-bound form. Thus, the final consequence is the activation of the replication cascade mediated by Mitogen-Activated Proteins (MAPs) and mitogen-activated protein kinases signals [3,18,19,20,21]. Other oncogenic mechanisms were also identified, such as the hindrance of cell adhesion, an increase in the reactive oxygen species concentration, and the inhibition of apoptosis [22,23,24,25].

The primary successful treatment strategy for CML is the use of tyrosine kinase inhibitors. Among these, imatinib was considered the gold-standard CML drug, being associated with a survival rate of over 80% at 10 years [26,27,28,29]. However, further mutations emerged and proved capable of disrupting its inhibitory and therapeutic activity [30,31].

There is a series of point mutations that result in different modulations of the structural profile of the enzyme and its response to a variety of drugs, including imatinib [30,32]. Among these, the T315I mutation is notorious for being associated with the worst cases of resistance by intensely disturbing the interaction network of first-choice inhibitors with the target enzyme [2,30]. This resistance conference is mainly associated with a strong stabilization of the active conformation of the BCR-ABL enzyme [33]. Access to the hydrophobic pocket is also significantly hampered, as isoleucine has a larger side chain than threonine [34], resulting in additional steric bumps.

To tackle these obstacles, ponatinib was designed as part of the third generation of tyrosine kinase inhibitors [35,36]. Its structure was explicitly designed to penetrate deep into the hydrophobic pocket of the target enzyme, resulting in the effective inhibition of the catalytic activity [34]. However, although ponatinib proved to be a valuable option for the treatment of CML when the T315I mutation is present, it was also shown to be significantly cardiotoxic [37,38].

Other tyrosine kinase inhibitors have been studied as potential candidates for the treatment of CML, including rebastinib, originally a tyrosine kinase inhibitor targeting the Tie2 series of protein kinases, which is associated with angiogenesis and is overexpressed in various tumors [39,40,41,42]. Although rebastinib was initially used for the inhibition of Tie2 kinase proteins, recent studies have shown its potential as an alternative drug for the treatment of CML [43,44,45], since it has been associated with effective inhibitory activity due to the presence of molecular scaffolds that interact with the ATP-binding region and the Glu282 and Arg386 residues in its structure [46]. This dual capacity is associated with an additional effect of conformational control that could assist in traditional competitive inhibition, resulting in highly potent inhibition of the T315I isoform of the BCR-ABL tyrosine kinase [46].

Given that attractive interactions, especially hydrogen bonds, are intimately related to the structure, control, regulation, and inhibition of kinase-type enzymes and other receptors [47,48,49,50,51,52,53], an in-depth study of rebastinib and ponatinib interactions with the BCR-ABL protein was performed to better understand and indicate different aspects in their electronic interactions with the target enzyme that may translate to their different therapeutic effects and inhibitory capacities. This context was achieved through full ONIOM optimizations of the entire protein complexes, as well as the Quantum Theory of Atoms in Molecules (QTAIM) and Natural Bond Orbital (NBO) analysis, which enabled intricate evaluations of large biological systems. Therefore, this study explores the electronic interaction of rebastinib and ponatinib with the BCR-ABL protein, focusing on the role of hydrogen bonding in the kinase-type enzyme structure.

## 2. Results and Discussion

The structures of ponatinib and rebastinib, which are deposited in PDB codes 3IK3 and 3QRJ, respectively, were used to validate the optimized B3LYP and docking structures. To assess the differences in the geometries, we calculated the root mean square deviation (RMSD) values of the docking and B3LYP geometries of ponatinib in relation to the PDB of 3IK3, which are 0.7766 and 1.0520 Å, respectively. In the case of rebastinib, the RMSD values are smaller. The RMSDs of the docking and B3LYP geometries in relation to the PDB of rebastinib in 3QRJ are 0.0098 and 0.5089 Å, respectively. The RMSD between docking and optimized B3LYP geometries for ponatinib is 0.9869, and for rebastinib, it is 0.5099. Therefore, RMSD values smaller than 2.0 Å were obtained, which is a reasonable threshold for ligand-binding conformations [54,55].

We used the optimized geometries of the complexes to evaluate the distances and angles of the main hydrogen bonds involving residues Glu286, Met318, and Asp381—more specifically, the ionic oxygen atoms from the lateral chains of the Glu286 and the Asp381 residues that interact with polar hydrogen atoms of the TKIs. These residues are described in the literature as important regulators of the active conformation of the BCR-ABL enzyme [46,56,57,58,59]. Therefore, a study of the the electronic structure of their interactions with ligands can point out important aspects for the inhibition of the target protein. Furthermore, the energetic aspects of the systems were evaluated—specifically, the energies of the obtained frontier orbitals.

**Figure 1:**
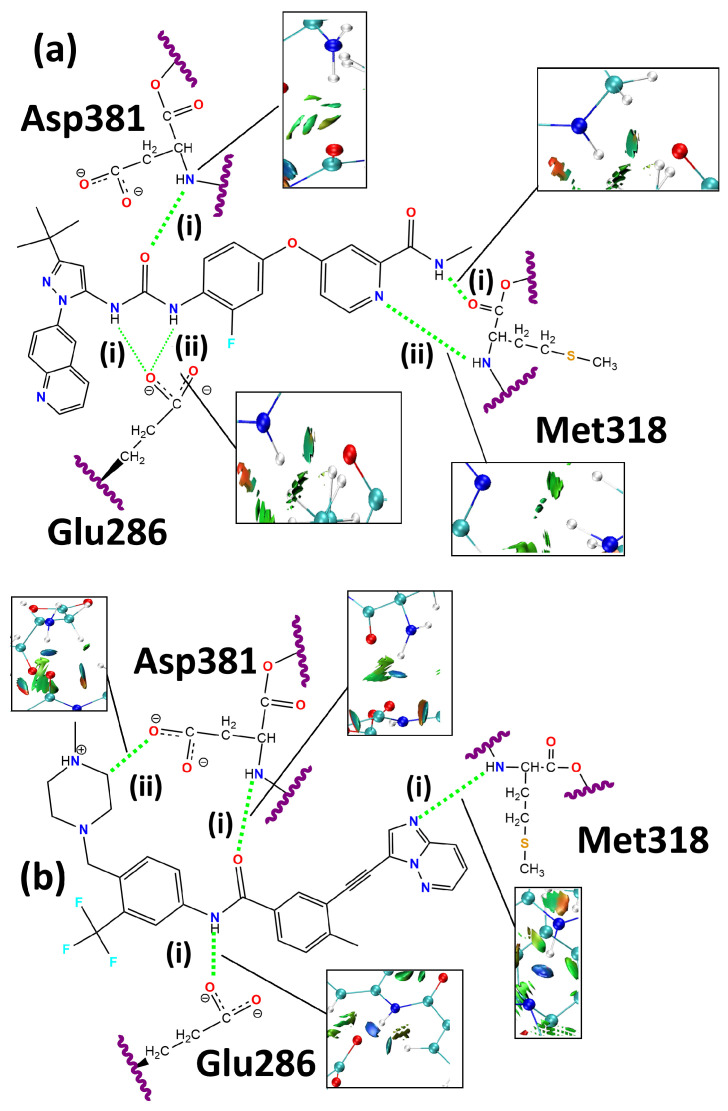
Two -dimensional structure and NCI formed between the (**a**) rebastinib and (**b**) ponatinib ligands and the BCR-ABL enzyme. The labels (i and ii) for the hydrogen bonding interaction types are also shown.

Figure 1 depicts the 2D structures and residues Glu286, Met318, and Asp381. The snapshots of NCI profiles of rebastinib and ponatinib are included. The complete NCI isosurface is shown in the Supplemental Materials.

Table 1 compiles the evaluated data of frontier molecular orbitals of BCR-ABL and BCR-ABL:ligand, which can be used to predict reactivity properties [60,61]. The gap value is the difference between the energy values of LUMO and HOMO ($E_{LUMO}-E_{HOMO}$). Gap values are dependent on the choice of density functional approximation (DFA), and it is well known that B3LYP underestimates the gap values [62]. However, the goal of this work is to determine the trends of frontier orbitals, which is effectively achieved with DFAs, not the gap’s absolute values [61,63,64,65,66,67].

It is important to note that the energies of the molecular orbitals, as well as the gap value, are only a pair of the indicators of stability and reactivity and that it is also necessary to consider other aspects related to the interaction of the protein with the studied ligands. The distances and angles of intermolecular interactions may indicate their intensity [68] and may also be related to the stabilization of the complex through this energy balance.

The B3LYP binding energy (BE) was calculated using the method of counterpoise correction proposed by Boys and Bernardi to evaluate the known Basis-Set Superposition Error (BSSE) [69]. The use of the BSSE is important to take into account for this small basis set used to compensate for the dispersion interaction [70]. The binding energy of rebastinib is 39.3 kcal·mol^−1^, which is smaller than the BE of ponatinib of 209.0 kcal·mol^−1^. However, in order to take into account the dispersion correction using the Grimme D3 dispersion correction, based on the B3LYP geometry, the BEs are 86.7 and 248.5 kcal·mol^−1^ for ponatinib and rebastinib, respectively. Moreover, since vibrational frequencies were used to characterize the minimum-energy DFT calculations, we also accounted for thermal corrections to the energies of these stable complexations. The Gibbs free energies for ponatinib and rebastinib are 81.5 kcal·mol^−1^ and 310.6 kcal·mol^−1^.

Table 2 shows the geometries of the key attractive interactions formed by rebastinib and ponatinib with the BCR-ABL enzyme. Many intermolecular interactions—especially hydrogen bonds—tend to achieve optimal geometries and, consequently, the highest stability at shorter distances and angles close to 180° [68].

After considering the initial information provided by the analysis of the geometry of each interaction, Table 3 yields further insight using the QTAIM and NBO data related to the interactions formed with Glu286, Met318, and Asp381. The interactions formed by ponatinib tend to be stronger. The hydrogen bond established with the Glu286 side chain surpasses both of its counterparts in the rebastinib complex, with the Laplacian values indicating that the electronic density of the interaction formed by ponatinib is closer to the atomic center.

According to Figure 1, the NCI profiles of rebastinib and ponatinib show different behaviors. It can be observed that ponatinib forms an additional and strong attractive interaction with the negatively charged side chain of Asp381, which is indicated by the dark-blue highlighted isosurface. Although rebastinib forms a hydrogen bond with Met318, it is suggested to be significantly weaker, as indicated by its isosurface. The absence of the contributing effects of an aromatic scaffold similar to that of ponatinib may be a cause for a weaker interaction [71]. As mentioned, both ligands also form attractive interactions with the Glu286 side chain.

Ponatinib has the highest gap value (Table 1) and is known as the best-performing molecule when applied to the BCR-ABL enzyme, which was expected, since its structure was designed for application to this protein [35]. Rebastinib also showed an increase in the gap value, but it was modest when compared to ponatinib while alternatively showing an increased value for the binding energy. This data corroborates its potential as an effective BCR-ABL inhibitor, which is described to form additional interactions with residues that ponatinib does not—specifically, Glu282 and Arg386 [46]. However, despite these extra interactions, it can be assumed, considering the evidence provided by the frontier molecular orbitals, that ponatinib may provide greater stabilization to the enzyme when examined through this method.

In relation to the docking structure, rebastinib improves its interaction distance (Table 2) with Glu286 through both hydrogen bonds identified along the ONIOM-optimized geometry. These interactions are depicted in Figure 1 as rebastinib:Glu286(i) and rebastinib:Glu286(ii), both involving the negatively charged carboxylate group of the Glu286 residue and hydrogen-bond donors in the form of the hydrogens bound to the nitrogens in the carbamide functional group of the rebastinib structure.

The rebastinib:Glu286(i) interaction had its length decreased by 0.119 Å while increasing the established angle by 10.46°. Alternatively, the rebastinib:Glu286(ii) interaction had its length decreased by 0.117 Å and its angle increased by 18.78°. An improvement in the geometric configuration of both interactions could be observed, indicating a strengthening of the attractive forces, given the influence of distances and angles on hydrogen-bond strengths [68]. The ONIOM-optimized lengths of the rebastinib:Glu286 interactions were shortened in relation to the docking and experimental data [46].

Ponatinib also forms an interaction with the carboxylate group of the Glu286 residue (Figure 1) using the N-H scaffold of its amide functional group as the hydrogen-bond donor. This interaction was labeled ponatinib:Glu286, and it reduces its distance to the side chain of the Glu286 residue throughout the geometry optimization by 0.041 Å while simultaneously increasing its interaction angle by 2.71°. It is worth noting that, similarly to what was observed in the interactions between the Glu286 residue and rebastinib, the distance and angle values became geometrically more favorable for strong hydrogen bonding, also indicating the formation of a stronger intermolecular interaction when compared to rebastinib due to the shorter length and larger angle. Additionally, both ligands were also shown to differ when their interactions with Asp381 were evaluated. Here, ponatinib reaches an equilibrium with an interaction distance only 0.017 Å larger than the one present in the starting geometry, and the interaction angle also decreased by a small value of 1.25°.

Another important point is the behavior of the attractive interactions formed between Met318 and the studied ligands. It can be observed that rebastinib forms two hydrogen bonds, both of which involve the polar backbone of the residue. The rebastinib:Met318(i) interaction is formed between the carbonyl group of the methionine structure and the N-H scaffold of the amide functional group of the ligand. Its length is reduced by 0.074 Å with optimization while also increasing the interacting angle by approximately 3°. A reduction in the interaction distance was not found in the case of the rebastinib:Met318(ii) interaction, which is formed between the amine function of the methionine residue and the nitrogen contained in the pyridine ring of rebastinib. This interaction shows an increase of 0.153 Å, while the angle also increased by 6.876°. The optimized structure interaction shows a geometry with an interacting angle closer to an ideal linear interaction and a shorter length compared to the crystallized rebastinib in complex with the BCR-ABL enzyme, specifically in the case of rebastinib:Met318(ii) [46].

The NCI isosurface for the rebastinib:Met318(ii) shown in Figure 1 is more intense compared to rebastinib:Met318(i), which can be associated with its geometry and the involvement of nitrogen in an aromatic ring, possibly increasing the attractive forces [71,72]. A similar effect may be associated with the hydrogen bond formed between the Met318 residue and ponatinib (ponatinib:Met318), which involves the amine functional group of the methionine backbone as a hydrogen-bond donor and nitrogen contained in the imidazopyridazine scaffold of the ligand. This interaction is well established as one of the most important in this system, as it provides access to the hydrophobic pocket of the BCR-ABL enzyme and ponatinib is particularly designed to favor this hydrogen bond [34,35]. This interaction increased its length by 0.002 Å and its angle by 0.25°, resulting in an optimized geometry that is more favorable for the formation of an intense hydrogen bond [68] than what is observed in both interactions involving the Met318 residue and rebastinib.

As for the interactions formed with the Asp381 residue in the DFG switch, it is possible to observe that rebastinib forms a single hydrogen bond, while ponatinib forms two. Rebastinib interacts with the Asp381 residue through the C=O scaffold of its carbamide functional group. This hydrogen bond was labeled rebastinib:Asp381, and its length increased by 0.196 Å in the ONIOM-optimized geometry. An increase was also observed in the interaction angle, which was 7.68° larger in the ONIOM-optimized geometry. Alternatively, ponatinib formed a conventional hydrogen bond through the oxygen of its amide functional group, which interacts with the amine function of the backbone of the Asp381 residue, ponatinib:Asp381(i). This interaction is found to be relatively conserved in the ONIOM-optimized geometry, with a modest increase in its length measuring 0.017 Å and a decrease of the interaction angle of 1.25°.

The additional interaction formed between ponatinib and the Asp381 residue, ponatinib:Asp381(ii), is an unconventional hydrogen bond established by the resulting attractive force between a partially charged methylene group in the 4-methylpiperazine ring and the carboxylate functional group in the side chain of the Asp381 residue. This interaction was made possible by the binding of the methylene group to a heavily polar nitrogen atom. As shown by Figure 1, this interaction is significantly intense and may be associated with effective inhibition of the BCR-ABL enzyme through the DFG switch [35,46,56].

Rebastinib shows an electron density distribution (Table 3) that is slightly more concentrated over the length of the interactions with Glu286 due to the smaller Laplacian value when compared to that of the ponatinib:Glu286 interaction, with a difference of 0.217 e·Å^−5^, as can be observed in Table 3. This difference indicates a stronger interaction that approaches a covalent interaction. This effect was shown to be modestly influential on the energy density (H^c^). The interaction formed with Glu286 by rebastinib and ponatinib has negative values, and following the concept of H^c^ introduced by Cremer and Kraka, negative values correspond to covalent interaction, while positive values show the electrostatic character of the weak interaction.

Alternatively, according to the NBO analysis, the smaller Laplacian value in the rebastinib:Glu286 interactions and consequent superior superposition of electronic density distribution along the interaction length could be associated with the higher value of the stabilization energy that the rebastinib:Glu286(ii) interaction shows when compared to ponatinib. However, the electron density observed in this interaction is smaller in rebastinib, albeit by a small margin.

Furthermore, according to Table 3, both NBO and BCP were found in the interactions formed with Met318 by ponatinib and rebastinib. As indicated by Figure 1 and Table 2, ponatinib forms a stronger interaction with the aforementioned residue, with a measured value for kinetic energy significantly larger than what was observed in both interactions between rebastinib and the Met318 residue, as well as almost double the electron density. The stabilization energy for the NBO in this interaction was measured as 19.96 kcal·mol^−1^, which is also significantly higher than that shown by rebastinib, which only reached 1.84 kcal·mol^−1^ in rebastinib:Met318(i) and 4.35 kcal·mol^−1^ in rebastinib:Met318(ii). All of the mentioned orbitals are of the LP-BD* type. This high energy difference can be explained by the intricate interplay involved in the electronic network of the interacting region, such as the fact that the LP-BD* hydrogen bond is enhanced by a larger orbital overlap and smaller orbital gap [71] when compared to its counterparts. It is associated with a more favorable orbital geometry under the lens of the Pauli repulsion [72,73]. It is also worth noting that the hydrogen-bond acceptor in this interaction is nitrogen contained within an intensely aromatic scaffold, an imidazopyridazine ring in conjugation with a triple bond, which is in resonance with another aromatic ring. This high aromaticity is a significant reinforcement of the hydrogen bond through π resonance assistance [71,74,75].

As for the interactions with the Asp381 residue, the BCP also indicates stronger interactions in the complex formed by ponatinib. Both interactions established by this ligand have higher kinetic energy than that of the one formed by rebastinib, with an associated higher electronic density. The Laplacian values indicate an electronic density more distributed over the interaction length in the rebastinib:Asp381 interaction when compared to ponatinib:Asp381(i) but not when compared to ponatinib:Asp381(ii). Additionally, two NBOs of importance were identified in the complex formed by ponatinib, while only one was identified in the in the complex formed with rebastinib. The NBOs identified in the former are of the LP-BD* type in both the ponatinib:Asp381(i) and ponatinib:Asp381(ii) interactions, with the first showing a stabilization energy of 7.11 kcal·mol^−1^, while the other has an associated energy of 3.95 kcal·mol^−1^. All of these NBOs showed a higher energy values when compared to the one in the rebastinib:Asp381 interaction; they are individually intense and are additionally subject to the cooperative effects that reinforce interactions in a network, resulting in a general attractive force that is greater than the sum of the contributions of each attractive interaction [71,76,77,78,79].

Considering all of the presented data, it is possible to observe that the binding mode of ponatinib as obtained by the AutoDock Vina algorithm is very close to that which results from further optimization via ONIOM calculations, and it is also the one associated with the formation of stronger attractive interactions, such as the hydrogen bonds formed with the Glu286, Met318, and Asp381 residues. For the Met318 residue specifically, the stabilization energy that could be identified in the interaction with ponatinib indicates a significantly intense and stable attractive force, which has also been described in the literature as one of the driving forces for the effectiveness of ponatinib through its influence on the affinity and consequent access to the hydrophobic pocket of the BCR-ABL enzyme [34,35,36,80]. Although rebastinib is a ligand with affinity for the same binding site as rebastinib, its capacity to interact with the Glu282 and Arg386 dyad, is located far from the Met318 site [46], may result in a weakening effect regarding the entrance into the hydrophobic pocket and may result in poorer performance regarding the competitive inhibition of the BCR-ABL enzyme.

**Figure 2:**
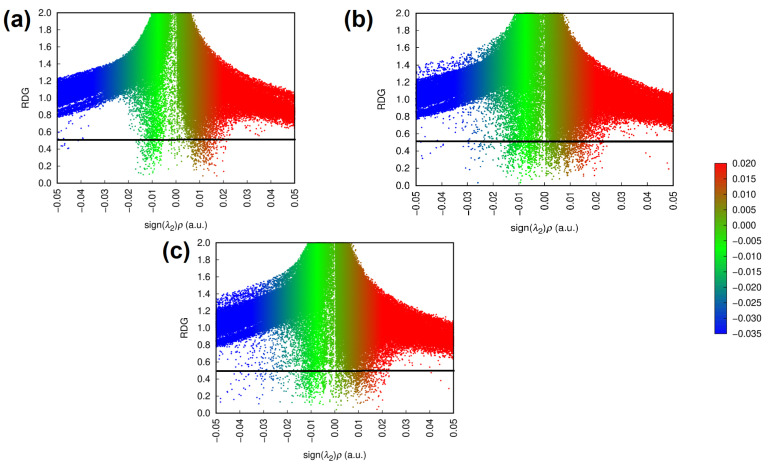
RDG plots representing the attractive and repulsive intermolecular interactions in the isolated BCR-ABL system (**a**) and in the presence of the rebastinib (**b**) and ponatinib (**c**) ligands as optimized via ONIOM.

The additional attractive interaction can also be seen in Figure 2, which shows the RDG plots of the interactions formed between the ligands and the target enzyme. In these plots, sign $\left(\lambda_{2}\right)$ represents the distribution of electronic density close to the formed interaction, with sign $(\lambda_{2})<0$ indicating high electronic densities and, therefore, attractive interactions [81,82,83,84].

According to Figure 2, the attractive interactions formed in the BCR-ABL + Ponatinib complex are more numerous and tend to be stronger. In contrast, the steric clashes and repulsive interactions indicated by the red spikes in the sign$(\lambda_{2})>0$ region are more similar to the profile exhibited by the isolated BCR-ABL enzyme, which may indicate that the interactions formed by ponatinib are less disruptive for the system as a whole, possibly due to its rigid and linear nature [35].

## 3. Methods

### 3.1. Docking Protocol

Aiming to obtain the starting geometries for the QM/MM calculations, the T315I isoform of the human BCR-ABL enzyme in the DFG-out conformation [46,59,85] was chosen as the target protein. Its crystal structure was obtained from the Protein Data Bank under code 3QRJ [46,86]. The structure was prepared for calculations with the removal of waters, the addition of hydrogens, the removal of doubled residues, and the repair of missing atoms through the AutoDock tool suite [87].

For the docking calculations, all rotatable bonds of the ligands were free, while the target protein was set to a rigid conformation. Considering that all ligands presented many rotatable bonds and elevated flexibility, the AutoDock Vina algorithm was used due to its adequacy for dealing with ligands with high degrees of flexibility [88], with the support of mathematical machine learning [88] techniques to compose its scoring function.

The search box was defined through the observation of the 3QRJ crystal structure, which is in complex with rebastinib (DCC-2036) [46]. The coordinates of this crystallized ligand were used to calculate a centroid, the origin point for the docking search, whose coordinates were set to x=−2.38327, y=12.76310, and z=12.57230 while measuring 22.25 × 23.00 × 25.25 Å in the *x*, *y*, and *z* axes, respectively. The exhaustiveness level of the search was set to 32 in an energy range of 3.0 kcal·mol^−1^ while searching for 1000 binding modes. These settings were validated through a redocking calculation using the crystallized DCC-2036 ligand, which provided a docking model with an RMSD value of 0.1438 Å, which is within the acceptable deviation range [55,89,90,91]. Visual analysis of the resulting docking conformations was performed using the Discovery Studio software package and the VMD suite [92,93]. The binding conformations obtained through the docking studies were used as the starting geometries for the ONIOM calculations.

### 3.2. ONIOM Calculations

The complexes comprising the BCR-ABL enzyme and the docked models of ponatinib and imatinib were then subjected to geometric optimizations using the two-layer ONIOM model [94] in order to find the equilibrium geometry. In each system, the Lys285, Glu286, Ala287, Ile315, Glu316, Phe317, Met318, Asp381, Phe382, and Gly383 residues were included in the high layer, along with the docked ligands, resulting in three different systems as follows:

1. The T315I isoform of the BCR-ABL enzyme in the absence of any ligands;
2. BCR-ABL(T315I) complexed with rebastinib;
3. BCR-ABL(T315I) complexed with ponatinib.

The Glu286, Ile315, Met318, and Asp381 residues are the key targets of modeling studies, as they are the most important in the inhibition of the BCR-ABL enzyme [34,56,85]. The electronic properties of the high layer were calculated according to the Density Functional Theory (DFT), using the B3LYP functional, along with the 6-31G(d) basis set [95,96]. The use of dispersion-corrected functionals is important for the strongly attractive, non-covalent interactions [97,98,99,100]. Nevertheless, the B3LYP hybrid functional is widely used in drug design studies due to its reasonable computational cost and reliable structural predictions [101,102,103,104,105]. The B3LYP results show reasonable agreement with respect to both the energy and geometries of hydrogen-bonded structures [105]. The hybrid approach of B3LYP and AMBER applying electronic embedding approximations has been used in the literature to obtain non-covalent results for the enzyme-inhibitor complex [106,107,108,109,110,111,112]. The optimized ONIOM and docking geometries of rebastinib and ponatinib are available in the Supplemental Materials.

The remaining residues and crystallographic waters were included in the low layer, whose properties were calculated through the AMBER force fields [113]. AMBER force-field studies are primarily based on this choice of basis set and functional: B3LYP and 6-31G(d) [114,115]. Despite the modest basis set used, the accuracy of the geometry for this basis set is reliable due to the large size and complexity of the studied system. Nevertheless, diffuse functions are required to yield appropriate energies. In this way, the possible size for the high-level ONIOM region is limited with large basis sets [116]. In contrast, using a large model adequate to improve the description of π−π interactions, B3LYP with a small basis set probably performs comparatively well for optimization geometry with less computational effort. However, it was found that the ONIOM approach using B3LYP/6-31G(d) improves the relative energies without achieving chemistry accuracy (less than 1 kcal·mol^−1^) [116,117,118,119,120]. For biological systems, the receptor binding energies were correctly assessed using ONIOM with B3LYP/6-31G(d), including BSSE [70,121]. Furthermore, based on B3LYP/6-31G(d) basis set under the ONIOM approach, the smallest median absolute deviation (MAD) was found for the proton and electron affinities of 1.29 kcal·mol^−1^ and 0.11 eV, respectively [122]. The inclusion of water molecules characterized an explicit solvation model for the calculations, which were performed using Gaussian16 software [123]. The calculated energy of the systems was obtained through Equation (1).

(1) $$E_{ONIOM(high:low)}=E_{high,model}+E_{low,real}-E_{low,model}$$

### 3.3. NCI, BCP, and NBO Analysis

Along with the calculated energies, the distances and angles of the hydrogen bonds formed with Glu286 and Asp381 were evaluated. The attractive charge interaction with the Asp381 side chain, when present, was also tracked. A qualitative analysis of Non-Covalent Interactions (NCIs) between the residues and ligands in the high-layer ONIOM-optimized structure was also performed using the Multiwfn 3.8 software package [124]. This analysis was complemented by a quantitative study of relevant Bond-Critical Points (BCPs) according to the Quantum Theory of Atoms In Molecules (QTAIM) [125] method using the AIMAll (Version 16.01.09) software package [126], which allowed for the evaluation of each interaction, mainly regarding the hydrogen bonds formed in the high layers of the complexes.

A Bond-Critical Point (BCP) can be characterized as a point in the electronic density space (ρ) where all the first derivatives of the density gradient ($\nabla_{\rho }$) are equal to zero [127,128]. The study of these points offers quantitative data for highly rigorous theoretical calculations, such as the strengths of intermolecular interactions [127].

Typically, the electron density at a bond-critical point ($\rho_{b}$) exceeds 1.35 e·Å^−3^ in covalent bonds, whereas it is less than 0.67 e·Å^−3^ in closed-shell interactions such as intermolecular interactions. These include a range of forces, from van der Waals interactions to hydrogen bonds and ionic interactions [127,128].

Additional insights can be gained by examining the Laplacian of the electron density at a given point ($\nabla^{2}\rho$). Negative values of the Laplacian indicate a concentration of electron density along the bond length, while positive values signify electron density concentration near the atomic nuclei. Consequently, in covalent bonds, $\nabla^{2}\rho <0$, whereas in intermolecular interactions, $\nabla^{2}\rho >0$. For example, the typical Laplacian value for a hydrogen bond is approximately 0.72 e·Å^−5^ [127,128]. Additionally, the Laplacian can be used to determine energy densities at the BCP. The Cremer–Kraka energy density (H^c^) is useful to indicate a non-covalent closed-shell interaction (electrostatic/dispersion type) for positive values, while H^c^ < 0 indicates a covalent interaction [129,130,131,132,133].

**Figure 3:**
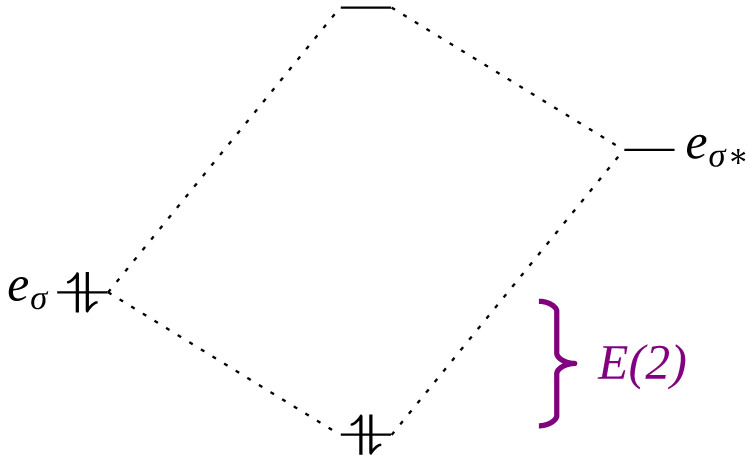
Representation of the stabilization energies (E2) of an LP-BD* type intermolecular interaction showing the bonding and antibonding (*) orbitals.

Finally, measurements of the donor–acceptor interactions were obtained through second-order perturbative analysis of Natural Bond Orbitals (NBOs). Natural Orbitals (NOs) are so-called due to their property of spontaneously emerging from the wave function as ideal descriptors. In the context of this study, the most important types of NBO are Lone Pair (LP), Bonding (BD) and Antibonding (BD*) NBOs. The analysis of NBOs can describe the results of orbital interactions, which are profoundly related to the stabilization energies (E(2)) of molecules and complexes through intermolecular interactions (Figure 3).

## 4. Conclusions

The present study was performed to provide a deeper understanding of the energetics involved in the binding modes of ponatinib and rebastinib regarding the mutant BCR-ABL enzyme according to refined QM/MM calculations. The analysis of docking conformations using AutoDock Vina through the lens of ONIOM provided important insights into the behavior of rebastinib and ponatinib when bound to the T315I isoform of the BCR-ABL enzyme. The aspects may be related to the difference between an effective and an ineffective inhibition model. Ponatinib was shown to form additional stronger and synergic hydrogen bonds, as well as to conserve them throughout the optimization of the docking geometry without significant deviations. QTAIM results and NBO analysis revealed the high indices involved in pertinent intermolecular interactions. The electronic properties—specifically, the LUMO−HOMO gap—of the BCR-ABL enzyme bound to ponatinib also showed indications of significant stabilization in the context of chemical reactivity, as suggested by the higher value calculated for the aforementioned gap. This decrease results in the inhibition of catalytic activity and enzyme activation due to more intense energetic barriers. Although rebastinib is described as a capable binder regarding the ATP-binding site of the enzyme, it was shown to do so to a lesser extent when compared to ponatinib, which can indicate that the former would be an inferior competitive inhibitor when compared to the latter. This behavior was expected, since ponatinib was specifically designed as a competitive inhibitor to the BCR-ABL enzyme, while rebastinib was originally aimed at the Tie2 family of enzymes and is described to act on the BCR-ABL enzyme not only through competitive inhibition.

## Figures and Tables

**Table 1:** Frontier molecular orbital energy values (eVs) of the BCR-ABL enzyme and its complexes formed with rebastinib and ponatinib according to AutoDock Vina and ONIOM calculations using the B3LYP functional with the 6-31G(d) basis set.

Parameter	BCR-ABL	BCR-ABL + Rebastinib	BCR-ABL + Ponatinib
HOMO	−3.393	−6.631	−3.193
LUMO	−3.244	−6.428	−0.883
Gap	0.149	0.203	2.310

**Table 2:** Docking and ONIOM-optimized geometries of the studied interactions of the complexes formed by the BCR-ABL enzyme with rebastinib and ponatinib. Data for hydrogen-bond distances and angles (See Figure 1 for the notation used).

Interaction	Interatomic Distance (Å)		Bond Angle (Degrees)	
	**Docking**	**ONIOM**	**Docking**	**ONIOM**
Rebastinib:Glu286 (i)	2.029	1.910	141.6	152.1
Rebastinib:Glu286 (ii)	2.040	1.923	132.5	151.3
Rebastinib:Met318 (i)	2.218	2.144	127.4	130.2
Rebastinib:Met318 (ii)	2.218	2.371	163.3	170.2
Rebastinib:Asp381 (i)	1.979	2.175	163.6	171.3
Ponatinib:Glu286 (i)	1.823	1.864	165.2	167.9
Ponatinib:Met318 (i)	1.922	1.924	171.2	171.5
Ponatinib:Asp381 (i)	1.888	1.905	170.2	168.9
Ponatinib:Asp381 (ii)	1.808	1.806	165.5	160.5

**Table 3:** QTAIM and NBO data of the intermolecular interactions formed by rebastinib and ponatinib bound to the BCR-ABL enzyme. Figure 1 shows the labels used (i and ii).

Interaction	Energy Density (a.u.)	Electron Density (e·Å^−3^)	Laplacian (e·Å^−5^)	E^(2)^ (kcal·mol^−1^)
Rebastinib:Glu286 (i)	−0.0016	0.206	2.087	8.38 ^(a)^
Rebastinib:Glu286 (ii)	−0.0016	0.206	2.087	14.09 ^(a)^
Rebastinib:Met318 (i)	0.0005	0.115	1.465	1.84 ^(b)^
Rebastinib:Met318 (ii)	0.0005	0.086	0.919	4.35 ^(c)^
Rebastinib:Asp381(i)	0.0001	0.101	1.199	2.47 ^(d)^
Ponatinib:Glu286 (i)	−0.0014	0.219	2.304	10.68 ^(a)^
Ponatinib:Met318 (i)	−0.0012	0.222	2.216	19.96 ^(c)^
Ponatinib:Asp381 (i)	−0.0001	0.182	2.229	7.11 ^(d)^
Ponatinib:Asp381 (ii)	−0.0026	0.285	2.914	3.95 ^(e)^

^(a)^ LP (Glu286)→ BD* (Rebastinib/Ponatinib); ^(b)^ LP (Met318) → BD* (Rebastinib); ^(c)^ LP (Rebastinib/Ponatinib) → BD* (Met318); ^(d)^ LP (Rebastinib/Ponatinib) → BD* (Asp381) ^(e)^ LP (Asp381) → BD* (Rebastinib/Ponatinib).

## Data Availability

The original contributions presented in this study are included in the article/Supplementary Materials. Further inquiries can be directed to the corresponding author.

## Supplementary Materials

The following supporting information can be downloaded at: https://www.mdpi.com/article/10.3390/molecules30204145/s1, Figure S1: Representation of the NCI established between the residues of the upper layer of the isolated Bcr-Abl enzyme (a) and in the presence of the ligands rebastinib (b) and ponatinib (c). Table S1. Optimized ONIOM and docking geometries of rebastinib. Table S2. Optimized ONIOM and docking geometries of ponatinib.

## References

[1] Sampaio M.M., Santos M.L.C., Marques H.S., Gonçalves V.L.d.S., Araújo G.R.L., Lopes L.W., Apolonio J.S., Silva C.S., Santos L.K.d.S., Cuzzuol B.R. (2021). Chronic myeloid leukemia-from the Philadelphia chromosome to specific target drugs: A literature review. World J. Clin. Oncol..

[2] Pricl S., Fermeglia M., Ferrone M., Tamborini E. (2005). T315I-mutated Bcr-Abl in chronic myeloid leukemia and imatinib: Insights from a computational study. Mol. Cancer Ther..

[3] Cilloni D., Saglio G. (2012). Molecular pathways: BCR-ABL. Clin. Cancer Res..

[4] Osman A.E., Deininger M.W. (2021). Chronic Myeloid Leukemia: Modern therapies, current challenges and future directions. Blood Rev..

[5] Rohrbacher M., Hasford J. (2009). Epidemiology of chronic myeloid leukaemia (CML). Best Pract. Res. Clin. Haematol..

[6] Zámečníkova A. (2010). Targeting the BCR-ABL tyrosine kinase in chronic myeloid leukemia as a model of rational drug design in cancer. Expert Rev. Hematol..

[7] Tosta Perez M., Herrera Belen L., Letelier P., Calle Y., Pessoa A., Farias J.G. (2023). l-Asparaginase as the gold standard in the treatment of acute lymphoblastic leukemia: A comprehensive review. Med. Oncol..

[8] Bernardo P.S., Lemos L.G.T., Moraes G.N.d., Maia R.C. (2020). Unraveling survivin expression in chronic myeloid leukemia: Molecular interactions and clinical implications. Blood Rev..

[9] Amarante-Mendes G.P., Rana A., Datoguia T.S., Hamerschlak N., Brumatti G. (2022). BCR-ABL1 Tyrosine Kinase Complex Signaling Transduction: Challenges to Overcome Resistance in Chronic Myeloid Leukemia. Pharmaceutics.

[10] Kang Z.J., Liu Y.F., Xu L.Z., Long Z.J., Huang D., Yang Y., Liu B., Feng J.X., Pan Y.J., Yan J.S. (2016). The Philadelphia chromosome in leukemogenesis. Chin. J. Cancer.

[11] Watt J.L., Page B.M. (1978). Reciprocal translocation and the Philadelphia chromosome. Hum. Genet..

[12] Minciacchi V.R., Kumar R., Krause D.S. (2021). Chronic Myeloid Leukemia: A Model Disease of the Past, Present and Future. Cells.

[13] Dobrovic A., Peters G.B., Ford J.H. (1991). Review: Molecular analysis of the Philadelphia chromosome. Chromosoma.

[14] Davulcu E.A., Pekerbas M., Karaca E., Durmaz B., Özsan N., Akın H., Saydam G. (2022). Complex karyotype with double Philadelphia chromosome and T315I mutation results in blastic phase and extensive extramedullary infiltration in a chronic myeloid leukemia patient. Cancer Genet..

[15] Reinhold U., Hennig E., Leiblein S., Niederwieser D., Deininger M.W.N. (2003). FISH for BCR-ABL on interphases of peripheral blood neutrophils but not of unselected white cells correlates with bone marrow cytogenetics in CML patients treated with imatinib. Leukemia.

[16] Dewald G.W., Wyatt W.A., Juneau A.L., Carlson R.O., Zinsmeister A.R., Jalal S.M., Spurbeck J.L., Silver R.T. (1998). Highly sensitive fluorescence in situ hybridization method to detect double BCR/ABL fusion and monitor response to therapy in chronic myeloid leukemia. Blood.

[17] Chopra R., Pu Q.Q., Elefanty A.G. (1999). Biology of BCR-ABL. Blood Rev..

[18] Malagrinò F., Puglisi E., Pagano L., Travaglini-Allocatelli C., Toto A. (2024). GRB2: A dynamic adaptor protein orchestrating cellular signaling in health and disease. Biochem. Biophys. Rep..

[19] Bar-Sagi D. (1994). The Sos (Son of sevenless) protein. Trends Endocrinol. Metab. TEM.

[20] Simanshu D.K., Nissley D.V., McCormick F. (2017). RAS Proteins and Their Regulators in Human Disease. Cell.

[21] Wei Z., Liu H.T. (2002). MAPK signal pathways in the regulation of cell proliferation in mammalian cells. Cell Res..

[22] Bedi A., Zehnbauer B.A., Barber J.P., Sharkis S.J., Jones R.J. (1994). Inhibition of apoptosis by BCR-ABL in chronic myeloid leukemia. Blood.

[23] Stein S.J., Baldwin A.S. (2011). NF-B suppresses ROS levels in BCR-ABL+ cells to prevent activation of JNK and cell death. Oncogene.

[24] Wertheim J.A., Forsythe K., Druker B.J., Hammer D., Boettiger D., Pear W.S. (2002). BCR-ABL-induced adhesion defects are tyrosine kinase-independent. Blood.

[25] Nakamura H., Takada K. (2021). Reactive oxygen species in cancer: Current findings and future directions. Cancer Sci..

[26] Sausville E.A. (2003). Imatinib for chronic myelogenous leukaemia: A 9 or 24 carat gold standard?. Lancet.

[27] Iqbal N., Iqbal N. (2014). Imatinib: A Breakthrough of Targeted Therapy in Cancer. Chemother. Res. Pract..

[28] Hochhaus A., Larson R.A., Guilhot F., Radich J.P., Branford S., Hughes T.P., Baccarani M., Deininger M.W., Cervantes F., Fujihara S. (2017). Long-Term Outcomes of Imatinib Treatment for Chronic Myeloid Leukemia. N. Engl. J. Med..

[29] Peng B., Lloyd P., Schran H. (2005). Clinical pharmacokinetics of imatinib. Clin. Pharmacokinet..

[30] Soverini S., Rosti G., Iacobucci I., Baccarani M., Martinelli G. (2011). Choosing the Best Second-Line Tyrosine Kinase Inhibitor in Imatinib-Resistant Chronic Myeloid Leukemia Patients Harboring Bcr-Abl Kinase Domain Mutations: How Reliable Is the IC50?. Oncologist.

[31] Volpe G., Panuzzo C., Ulisciani S., Cilloni D. (2009). Imatinib resistance in CML. Cancer Lett..

[32] Cang S., Liu D. (2008). P-loop mutations and novel therapeutic approaches for imatinib failures in chronic myeloid leukemia. J. Hematol. Oncol..

[33] Rosari F., Minutolo F., Orciuolo E. (2018). Past, present, and future of Bcr-Abl inhibitors: From chemical development to clinical efficacy. J. Hematol. Oncol..

[34] Pandrala M., Bruyneel A.A.N., Hnatiuk A.P., Mercola M., Malhotra S.V. (2022). Designing Novel BCR-ABL Inhibitors for Chronic Myeloid Leukemia with Improved Cardiac Safety. J. Med. Chem..

[35] O’Hare T., Shakespeare W.C., Zhu X., Eide C.A., Rivera V.M., Wang F., Adrian L.T., Zhou T., Huang W.S., Xu Q. (2009). AP24534, a Pan-BCR-ABL Inhibitor for Chronic Myeloid Leukemia, Potently Inhibits the T315I Mutant and Overcomes Mutation-Based Resistance. Cancer Cell.

[36] Tanneeru K., Guruprasad L. (2013). Ponatinib Is a Pan-BCR-ABL Kinase Inhibitor: MD Simulations and SIE Study. PLoS ONE.

[37] Marto J.P., Strambo D., Livio F., Michel P. (2021). Drugs Associated With Ischemic Stroke: A Review for Clinicians. Stroke.

[38] Tousif S., Singh A.P., Umbarkar P., Galindo C., Wheeler N., Toro Cora A., Zhang Q., Prabhu S.D., Lal H. (2023). Ponatinib Drives Cardiotoxicity by S100A8/A9-NLRP3-IL-1*β* Mediated Inflammation. Circ. Res..

[39] Zadeh G., Qian B., Okhowat A., Sabha N., Kontos C.D., Guha A. (2004). Targeting the Tie2/Tek Receptor in Astrocytomas. Am. J. Pathol..

[40] Huang H., Bhat A., Woodnutt G., Lappe R. (2010). Targeting the ANGPT–TIE2 pathway in malignancy. Nat. Rev. Cancer.

[41] Hasenstein J.R., Kasmerchak K., Buehler D., Hafez G.R., Cleary K., Moody J.S., Kozak K.R. (2012). Efficacy of Tie2 receptor antagonism in Angiosarcoma. Neoplasia.

[42] Martin V., Liu D., Fueyo J., Gomez-Manzano C. (2008). Tie2: A journey from normal angiogenesis to cancer and beyond. Histol. Histopathol..

[43] Ashaq M.S., Zhou Q., Li Z., Zhao B. (2024). Novel targeted therapies in chronic myeloid leukemia. Pharm. Sci. Adv..

[44] Cortes J., Talpaz M., Smith H.P., Snyder D.S., Khoury J., Bhalla K.N., Pinilla-Ibarz J., Larson R., Mitchell D., Wise S.C. (2017). Phase 1 dose-finding study of rebastinib (DCC-2036) in patients with relapsed chronic myeloid leukemia and acute myeloid leukemia. Haematologica.

[45] Mojtahedi H., Yazdanpanah N., Rezaei N. (2021). Chronic myeloid leukemia stem cells: Targeting therapeutic implications. Stem Cell Res. Ther..

[46] Chan W.W., Wise S.C., Kaufman M.D., Ahn Y.M., Ensinger C.L., Haack T., Hood M.M., Jones J., Lord J.W., Lu W.P. (2011). Conformational Control Inhibition of the BCR-ABL1 Tyrosine Kinase, Including the Gatekeeper T315I Mutant, by the Switch-Control Inhibitor DCC-2036. Cancer Cell.

[47] Antolíková E., Žáková L., Turkenburg J.P., Watson C.J., Hančlová I., Šanda M., Cooper A., Kraus T., Brzozowski A.M., Jiráček J. (2011). Non-equivalent role of inter- and intramolecular hydrogen bonds in the insulin dimer interface. J. Biol. Chem..

[48] Wootten D., Reynolds C.A., Koole C., Smith K.J., Mobarec J.C., Simms J., Quon T., Coudrat T., Furness S.G., Miller L.J. (2016). A hydrogen-bonded polar network in the core of the glucagon-like peptide-1 receptor is a fulcrum for biased agonism: Lessons from class B crystal structuress. Mol. Pharmacol..

[49] Venugopal P.P., Das B.K., Soorya E., Chakraborty D. (2020). Effect of hydrophobic and hydrogen bonding interactions on the potency of ß-alanine analogs of G-protein coupled glucagon receptor inhibitors. Proteins Struct. Funct. Bioinform..

[50] Hubbard R.E., Kamran Haider M. (2010). Hydrogen Bonds in Proteins: Role and Strength. Encyclopedia of Life Sciences.

[51] Pace C.N., Fu H., Fryar K.L., Landua J., Trevino S.R., Schell D., Thurlkill R.L., Imura S., Scholtz J.M., Gajiwala K. (2014). Contribution of hydrogen bonds to protein stability. Protein Sci..

[52] Xing L., Klug-Mcleod J., Rai B., Lunney E.A. (2015). Kinase hinge binding scaffolds and their hydrogen bond patterns. Bioorganic Med. Chem..

[53] Li G.C., Srivastava A.K., Kim J., Taylor S.S., Veglia G. (2015). Mapping the Hydrogen Bond Networks in the Catalytic Subunit of Protein Kinase A Using H/D Fractionation Factors. Biochemistry.

[54] Ding Y., Fang Y., Moreno J., Ramanujam J., Jarrell M., Brylinski M. (2016). Assessing the similarity of ligand binding conformations with the Contact Mode Score. Comput. Biol. Chem..

[55] Castro-Alvarez A., Costa A.M., Vilarrasa J. (2017). The Performance of several docking programs at reproducing protein-macrolide-like crystal structures. Molecules.

[56] Reddy E.P., Aggarwal A.K. (2012). The Ins and Outs of Bcr-Abl Inhibition. Genes Cancer.

[57] Rocha K.M., Nascimento É.C., Martins J.B. (2021). Investigation on the interaction behavior of afatinib, dasatinib, and imatinib docked to the BCR-ABL protein. J. Mol. Model..

[58] Rocha K.M.L., Nascimento E.C.M., de Jesus R.C.C., Martins J.B.L. (2024). In Silico Molecular Modeling of Four New Afatinib Derived Molecules Targeting the Inhibition of the Mutated Form of BCR-ABL T315I. Molecules.

[59] Pereira W.A., Nascimento É.C.M., Martins J.B.L. (2022). Electronic and structural study of T315I mutated form in DFG-out conformation of BCR-ABL inhibitors. J. Biomol. Struct. Dyn..

[60] Miar M., Shiroudi A., Pourshamsian K., Oliaey A.R., Hatamjafari F. (2021). Theoretical investigations on the HOMO–LUMO gap and global reactivity descriptor studies, natural bond orbital, and nucleus-independent chemical shifts analyses of 3-phenylbenzo[d]thiazole-2(3H)-imine and its para-substituted derivatives: Solvent and substituent effects. J. Chem. Res..

[61] Yu J., Su N.Q., Yang W. (2022). Describing Chemical Reactivity with Frontier Molecular Orbitalets. JACS Au.

[62] Bryenton K.R., Adeleke A.A., Dale S.G., Johnson E.R. (2022). Delocalization error: The greatest outstanding challenge in density-functional theory. WIREs Comput. Mol. Sci..

[63] Jursic B. (2000). A B3LYP hybrid density functional theory study of structural properties, energies, and heats of formation for silicon–hydrogen compounds. J. Mol. Struct. THEOCHEM.

[64] Costa R.J., Castro E.A.S., Politi J.R.S., Gargano R., Martins J.B.L. (2019). Methanol, ethanol, propanol, and butanol adsorption on H-ZSM-5 zeolite: An ONIOM study. J. Mol. Model..

[65] de Souza Farias S.A., da Costa K.S., Martins J.B.L. (2021). Analysis of Conformational, Structural, Magnetic, and Electronic Properties Related to Antioxidant Activity: Revisiting Flavan, Anthocyanidin, Flavanone, Flavonol, Isoflavone, Flavone, and Flavan-3-ol. ACS Omega.

[66] Nascimento E.C.M., Martins J.B.L. (2010). Electronic structure and PCA analysis of covalent and non-covalent acetylcholinesterase inhibitors. J. Mol. Model..

[67] Nascimento L.A., Nascimento É.C., Martins J.B. (2022). In silico study of tacrine and acetylcholine binding profile with human acetylcholinesterase: Docking and electronic structure. J. Mol. Model..

[68] Herschlag D., Pinney M.M. (2018). Hydrogen Bonds: Simple after All?. Biochemistry.

[69] Boys S.F., Bernardi F. (1970). The calculation of small molecular interactions by the differences of separate total energies. Some procedures with reduced errors. Mol. Phys..

[70] Kruse H., Goerigk L., Grimme S. (2012). Why the standard B3LYP/6-31G* model chemistry should not be used in DFT calculations of molecular thermochemistry: Understanding and correcting the problem. J. Org. Chem..

[71] van der Lubbe S.C., Guerra C.F. (2019). The Nature of Hydrogen Bonds: A Delineation of the Role of Different Energy Components on Hydrogen Bond Strengths and Lengths. Chem. Asian J..

[72] van der Lubbe S.C., Guerra C.F. (2017). Hydrogen-Bond Strength of CC and GG Pairs Determined by Steric Repulsion: Electrostatics and Charge Transfer Overruled. Chem. A Eur. J..

[73] Mao Y., Horn P.R., Head-Gordon M. (2017). Energy decomposition analysis in an adiabatic picture. Phys. Chem. Chem. Phys..

[74] Gilli G., Bellucci F., Ferretti V., Bertolasi V. (1989). Evidence for resonance-assisted hydrogen bonding from crystal-structure correlations on the enol form of the .beta.-diketone fragment. J. Am. Chem. Soc..

[75] Bertolasi V., Gilli P., Ferretti V., Gilli G. (1991). Evidence for resonance-assisted hydrogen bonding. 2. Intercorrelation between crystal structure and spectroscopic parameters in eight intramolecularly hydrogen bonded 1,3-diaryl-1,3-propanedione enols. J. Am. Chem. Soc..

[76] Mahadevi A.S., Sastry G.N. (2016). Cooperativity in Noncovalent Interactions. Chem. Rev..

[77] Kar T., Scheiner S. (2004). Comparison of cooperativity in CH...O and OH...O hydrogen bonds. J. Phys. Chem. A.

[78] Chen Y.F., Dannenberg J.J. (2006). Cooperative 4-pyridone H-bonds with extraordinary stability. A DFT molecular orbital study. J. Am. Chem. Soc..

[79] Kobko N., Paraskevas L., Del Rio E., Dannenberg J.J. (2001). Cooperativity in amide hydrogen bonding chains: Implications for protein-folding models. J. Am. Chem. Soc..

[80] Mahmoudi Gomari M., Rostami N., Ghodrati A., Hernandez Y., Fadaie M., Sadegh Eslami S., Tarighi P. (2021). Implementation of docking, molecular dynamics and free energy to investigate drug potency of novel BCR-ABLT315I inhibitors as an alternative to ponatinib. Comput. Toxicol..

[81] Contreras-García J., Yang W., Johnson E.R. (2011). Analysis of Hydrogen-Bond Interaction Potentials from the Electron Density: Integration of Noncovalent Interaction Regions. J. Phys. Chem. A.

[82] Castro T.S., Martins G.F., de Alcântara Morais S.F., Ferreira D.A.C. (2023). Aromaticity of Cope and Claisen rearrangements. Theor. Chem. Accounts.

[83] Martins G.F., Castro T.S., Ferreira D.A.C. (2023). Theoretical investigation of anion perfluorocubane. J. Mol. Model..

[84] Ponnuchamy V., Sandak A., Sandak J. (2020). Multiscale modelling investigation of wood modification with acetic anhydride †. Phys. Chem. Chem. Phys..

[85] Zhou T., Commodore L., Huang W.S., Wang Y., Thomas M., Keats J., Xu Q., Rivera V.M., Shakespeare W.C., Clackson T. (2011). Structural Mechanism of the Pan-BCR-ABL Inhibitor Ponatinib (AP24534): Lessons for Overcoming Kinase Inhibitor Resistance. Chem. Biol. Drug Des..

[86] Berman H.M., Westbrook J., Feng Z., Gilliland G., Bhat T.N., Weissig H., Shindyalov I.N., Bourne P.E. (2000). The Protein Data Bank. Nucleic Acids Res..

[87] Morris G.M., Huey R., Lindstrom W., Sanner M.F., Belew R.K., Goodsell D.S., Olson A.J. (2009). AutoDock4 and AutoDockTools4: Automated docking with selective receptor flexibility. J. Comput. Chem..

[88] Trott O., Olson A.J. (2010). AutoDock Vina: Improving the speed and accuracy of docking with a new scoring function, efficient optimization, and multithreading. J. Comput. Chem..

[89] Shivanika C., Deepak Kumar S., Ragunathan V., Tiwari P., Sumitha A., Brindha Devi P. (2022). Molecular docking, validation, dynamics simulations, and pharmacokinetic prediction of natural compounds against the SARS-CoV-2 main-protease. J. Biomol. Struct. Dyn..

[90] Ramírez D., Caballero J. (2018). Is It Reliable to Take the Molecular Docking Top Scoring Position as the Best Solution without Considering Available Structural Data?. Molecules.

[91] Silva Andrade B., Ghosh P., Barh D., Tiwari S., José Santana Silva R., Rodrigues de Assis Soares W., Silva Melo T., Santos Freitas A., González-Grande P., Sousa Palmeira L. (2020). Computational screening for potential drug candidates against the SARS-CoV-2 main protease. F1000Research.

[92] Biovia R.D.S. (2017). Discovery Studio Modeling Environment.

[93] Humphrey W., Dalke A., Schulten K. (1996). VMD: Visual molecular dynamics. J. Mol. Graph..

[94] Chung L.W., Sameera W.M., Ramozzi R., Page A.J., Hatanaka M., Petrova G.P., Harris T.V., Li X., Ke Z., Liu F. (2015). The ONIOM Method and Its Applications. Chem. Rev..

[95] Ditchfield R., Hehre W.J., Pople J.A. (1971). Self-consistent molecular-orbital methods. IX. An extended gaussian-type basis for molecular-orbital studies of organic molecules. J. Chem. Phys..

[96] Lee C., Yang W., Parr R.G. (1988). Development of the Colic-Salvetti correlation-energy formula into a functional of the electron density. Phys. Rev. B.

[97] Spicher S., Caldeweyher E., Hansen A., Grimme S. (2021). Benchmarking London dispersion corrected density functional theory for noncovalent ion–*π* interactions. Phys. Chem. Chem. Phys..

[98] Liao M.S., Huang M.J., Watts J.D. (2012). Assessment of dispersion corrections in DFT calculations on large biological systems. Mol. Phys..

[99] Körzdörfer T., Sears J.S., Sutton C., Brédas J.L. (2011). Long-range corrected hybrid functionals for *π*-conjugated systems: Dependence of the range-separation parameter on conjugation length. J. Chem. Phys..

[100] Szczepanik D.W., Solà M., Andrzejak M., Pawełek B., Dominikowska J., Kukułka M., Dyduch K., Krygowski T.M., Szatylowicz H. (2017). The role of the long-range exchange corrections in the description of electron delocalization in aromatic species. J. Comput. Chem..

[101] Guan H., Sun H., Zhao X. (2025). Application of Density Functional Theory to Molecular Engineering of Pharmaceutical Formulations. Int. J. Mol. Sci..

[102] Ouma R.B.O., Ngari S.M., Kibet J.K. (2024). A review of the current trends in computational approaches in drug design and metabolism. Discov. Public Health.

[103] Lu L., Hu H., Hou H., Wang B. (2013). An improved B3LYP method in the calculation of organic thermochemistry and reactivity. Comput. Theor. Chem..

[104] Ye N., Yang Z., Liu Y. (2022). Applications of density functional theory in COVID-19 drug modeling. Drug Discov. Today.

[105] Lozynski M., Rusinska-Roszak D., Mack H.G. (1998). Hydrogen Bonding and Density Functional Calculations: The B3LYP Approach as the Shortest Way to MP2 Results. J. Phys. Chem. A.

[106] Schmidt T.C., Welker A., Rieger M., Sahu P.K., Sotriffer C.A., Schirmeister T., Engels B. (2014). Protocol for Rational Design of Covalently Interacting Inhibitors. ChemPhysChem.

[107] Mihalovits L.M., Ferenczy G.G., Keserű G.M. (2022). The role of quantum chemistry in covalent inhibitor design. Int. J. Quantum Chem..

[108] Tóth L., Muszbek L., Komáromi I. (2013). Mechanism of the irreversible inhibition of human cyclooxygenase-1 by aspirin as predicted by QM/MM calculations. J. Mol. Graph. Model..

[109] Cuadrado C., Daranas A.H., Sarotti A.M. (2022). May the Force (Field) Be with You: On the Importance of Conformational Searches in the Prediction of NMR Chemical Shifts. Mar. Drugs.

[110] Yildiz I., Yildiz B.S. (2022). Computational Analysis of Histone Deacetylase 10 Mechanism by the ONIOM Method: A Complementary Approach to X-ray and Kinetics Studies. ACS Omega.

[111] Kar R.K. (2023). Benefits of hybrid QM/MM over traditional classical mechanics in pharmaceutical systems. Drug Discov. Today.

[112] Potier N., Barth P., Tritsch D., Biellmann J.F., Van Dorsselaer A. (1997). Study of non-covalent enzyme-inhibitor complexes of aldose reductase by electrospray mass spectrometry. Eur. J. Biochem..

[113] Ponder J.W., Case D.A. (2003). Force Fields for Protein Simulations. Adv. Protein Chem..

[114] Momany F., Willett J. (2000). Computational studies on carbohydrates: In vacuo studies using a revised AMBER force field, AMB99C, designed for *α*-(1→4) linkages. Carbohydr. Res..

[115] Freindorf M., Shao Y., Furlani T.R., Kong J. (2005). Lennard–Jones parameters for the combined QM/MM method using the B3LYP/6-31G*/AMBER potential. J. Comput. Chem..

[116] Kellie J.L., Wetmore S.D. (2013). Selecting DFT methods for use in optimizations of enzyme active sites: Applications to ONIOM treatments of DNA glycosylases. Can. J. Chem..

[117] Lundberg M., Sasakura Y., Zheng G., Morokuma K. (2010). Case studies of ONIOM(DFT:DFTB) and ONIOM(DFT:DFTB:MM) for enzymes and enzyme mimics. J. Chem. Theory Comput..

[118] Sharma H., Raju B., Narendra G., Motiwale M., Sharma B., Verma H., Silakari O. (2023). QM/MM studies on enzyme catalysis and insight into designing of new inhibitors by ONIOM approach: Recent update. ChemistrySelect.

[119] Nadia L., Djameleddine K., Rayenne D. (2014). Theoretical study of the inclusion processes of octopamine with *β*-cyclodextrin: PM6, ONIOM, and NBO analysis. Comptes Rendus. Chim..

[120] Tantirungrotechai Y., Roddecha S., Punyain K., Toochinda P. (2009). Assessment of mixed basis set and ONIOM methods on the activation energy of ring opening reactions of substituted cyclobutenes. J. Mol. Struct. THEOCHEM.

[121] Piyaauksornsak S., Tangthongkul T., Wanbayor R., Wanno B., Ruangpornvisuti V. (2009). Molecular structures of 8,8’-dithioureido-2,2’-binaphthalene derivatives and their anions recognition: An ONIOM investigation. Struct. Chem..

[122] Heerdt G., Morgon N.H. (2012). Theoretical study of thermochemical properties using composite methods adapted to ONIOM. J. Braz. Chem. Soc..

[123] Frisch M.J., Trucks G.W., Schlegel H.B., Scuseria G.E., Robb M.A., Cheeseman J.R., Scalmani G., Barone V., Petersson G.A., Nakatsuji H. (2016). Gaussian˜16 Revision C.01.

[124] Lu T., Chen F. (2012). Multiwfn: A multifunctional wavefunction analyzer. J. Comput. Chem..

[125] Bader R.F.W. (1991). A quantum theory of molecular structure and its applications. Chem. Rev..

[126] Keith T. (2019). AIMAll.

[127] Matta C.F., Lombardi O., Jaimes Arriaga J. (2020). Two-step emergence: The quantum theory of atoms in molecules as a bridge between quantum mechanics and molecular chemistry. Found. Chem..

[128] Oliveira B.G., Araújo R.C., Ramos M.N. (2010). The QTAIM Molecular Topology and the Quantum-Mechanical Description of Hydrogen Bonds and Dihydrogen Bonds. Quim. Nova.

[129] Oliveira V., Kraka E. (2017). Systematic Coupled Cluster Study of Noncovalent Interactions Involving Halogens, Chalcogens, and Pnicogens. J. Phys. Chem. A.

[130] Cremer D., Kraka E. (1984). Chemical Bonds without Bonding Electron Density — Does the Difference Electron-Density Analysis Suffice for a Description of the Chemical Bond?. Angew. Chem. Int. Ed. Engl..

[131] Gibbs G.V., Cox D.F., Crawford T.D., Rosso K.M., Ross N.L., Downs R.T. (2006). Classification of metal-oxide bonded interactions based on local potential- and kinetic-energy densities. J. Chem. Phys..

[132] Martins J.B., Quintino R.P., Politi J.R.d.S., Sethio D., Gargano R., Kraka E. (2020). Computational analysis of vibrational frequencies and rovibrational spectroscopic constants of hydrogen sulfide dimer using MP2 and CCSD(T). Spectrochim. Acta Part A Mol. Biomol. Spectrosc..

[133] Freindorf M., Kraka E., Cremer D. (2012). A comprehensive analysis of hydrogen bond interactions based on local vibrational modes. Int. J. Quantum Chem..

