# Supplementary material for: Under ONIOM Layers: Analysis of BCR-ABL Enzyme Inhibitors Through Bond-Critical Points and Natural Orbitals
Source: Molecules. 2025 Oct 21;30(20):4145. doi: 10.3390/molecules30204145 (PMC12566149; doi:10.3390/molecules30204145)
Supplement: Supplementary file 1 [file molecules-30-04145-s001.zip › molecules-3907037-supplementary.pdf]

Under ONIOM Layers: Analysis of BCR-ABL Enzyme Inhibitors Through Bond Critical Points and Natural Orbitals

Kelvyn M. L. Rocha [1] ,Érica C. M. Nascimento [2] and João B. L. Martins [1,2] *
1 Department of Pharmacy, Faculty of Health Sciences, University of Brasilia, Brasilia, DF, Brazil;
2 Laboratory of Computational Chemistry, Institute of Chemistry, University of Brasilia, Brasilia, DF, Brazil;
ericacristinamoreno@gmail.com
* Correspondence: lopes@unb.br

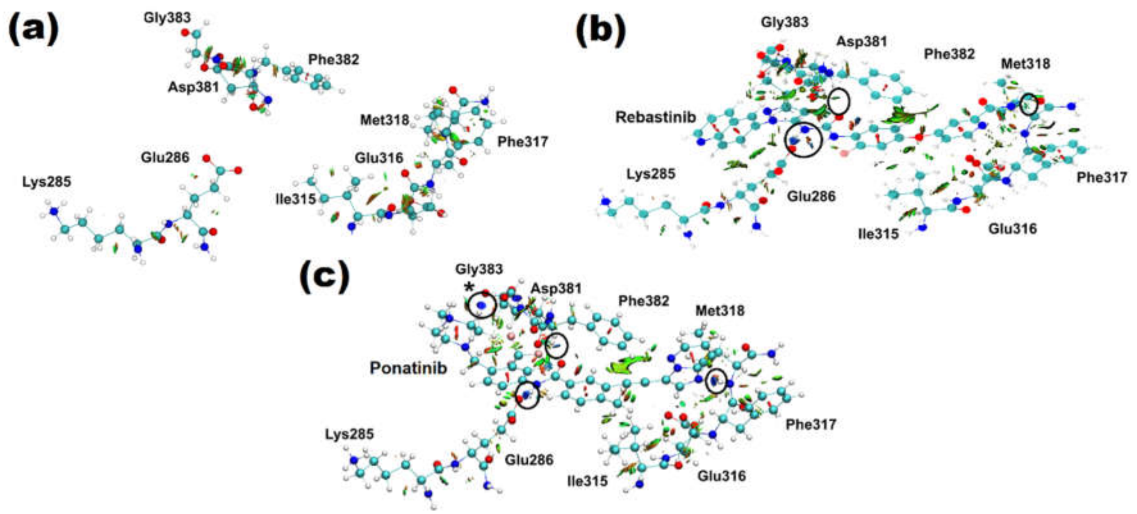

Figure S1: Representation of the NCI established between the residues of the upper layer of the isolated Bcr-Abl enzyme (a) and in the presence of the ligands rebastinib (b) and ponatinib (c)

Table S1. Optimized ONIOM and docking geometries of rebastinib.

Docking					X	Y	Z		ONIOM					X	Y	Z	
HETATM	1	C1	RES	1	-0.831	7.859	13.442	C	HETATM	1	C1	RES	1	-0.648	7.724	13.529	C
HETATM	2	C2	RES	1	-0.938	6.625	14.094	C	HETATM	2	C2	RES	1	-0.915	6.730	14.507	C
HETATM	3	C3	RES	1	-1.702	5.578	13.598	C	HETATM	3	C3	RES	1	-1.545	5.558	14.157	C
HETATM	4	C4	RES	1	-2.399	5.723	12.423	C	HETATM	4	C4	RES	1	-1.948	5.329	12.812	C
HETATM	5	C5	RES	1	-2.307	6.924	11.725	C	HETATM	5	C5	RES	1	-1.646	6.326	11.830	C
HETATM	6	C6	RES	1	-1.528	7.958	12.236	C	HETATM	6	C6	RES	1	-0.981	7.525	12.206	C
HETATM	7	C7	RES	1	-3.800	4.850	10.785	C	HETATM	7	C7	RES	1	-2.931	3.970	11.244	C
HETATM	8	N8	RES	1	-3.132	4.700	11.945	N	HETATM	8	N8	RES	1	-2.586	4.157	12.508	N
HETATM	9	C9	RES	1	-2.990	7.102	10.532	C	HETATM	9	C9	RES	1	-2.004	6.059	10.481	C
HETATM	10	C10	RES	1	-3.760	6.046	10.056	C	HETATM	10	C10	RES	1	-2.664	4.886	10.194	C
HETATM	11	C11	RES	1	-4.217	20.152	13.175	C	HETATM	11	C11	RES	1	-4.203	20.293	13.113	C
HETATM	12	N12	RES	1	-4.388	21.021	12.152	N	HETATM	12	N12	RES	1	-4.389	21.195	12.135	N
HETATM	13	C13	RES	1	-4.851	20.571	10.960	C	HETATM	13	C13	RES	1	-4.845	20.719	10.955	C
HETATM	14	C14	RES	1	-5.205	19.238	10.774	C	HETATM	14	C14	RES	1	-5.159	19.379	10.744	C
HETATM	15	C15	RES	1	-5.039	18.336	11.806	C	HETATM	15	C15	RES	1	-4.976	18.457	11.783	C
HETATM	16	C16	RES	1	-4.545	18.803	13.029	C	HETATM	16	C16	RES	1	-4.461	18.927	12.994	C
HETATM	17	C17	RES	1	-2.803	14.647	12.718	C	HETATM	17	C17	RES	1	-2.906	14.703	12.756	C
HETATM	18	C18	RES	1	-3.370	15.832	12.267	C	HETATM	18	C18	RES	1	-3.475	15.944	12.469	C
HETATM	19	C19	RES	1	-4.760	15.901	12.048	C	HETATM	19	C19	RES	1	-4.791	16.029	12.029	C
HETATM	20	C20	RES	1	-5.539	14.774	12.264	C	HETATM	20	C20	RES	1	-5.543	14.860	11.900	C
HETATM	21	C21	RES	1	-4.965	13.595	12.720	C	HETATM	21	C21	RES	1	-4.952	13.644	12.196	C
HETATM	22	C22	RES	1	-3.597	13.504	12.942	C	HETATM	22	C22	RES	1	-3.620	13.498	12.621	C
HETATM	23	C23	RES	1	1.794	9.676	14.589	C	HETATM	23	C23	RES	1	1.852	9.950	14.490	C
HETATM	24	C24	RES	1	0.892	10.742	14.494	C	HETATM	24	C24	RES	1	0.858	10.925	14.252	C
HETATM	25	C25	RES	1	-0.296	10.181	14.024	C	HETATM	25	C25	RES	1	-0.270	10.214	13.873	C

HETATM	26	N26	RES	1	-0.049	8.845	13.898	N
HETATM	27	N27	RES	1	1.275	8.497	14.206	N
HETATM	28	N28	RES	1	-1.547	10.678	13.761	N
HETATM	29	C29	RES	1	-1.847	11.949	13.429	C
HETATM	30	N30	RES	1	-3.141	12.300	13.364	N
HETATM	31	O31	RES	1	-0.995	12.768	13.183	O
HETATM	32	O32	RES	1	-5.394	17.030	11.577	O
HETATM	33	F33	RES	1	-5.751	12.529	12.972	F
HETATM	34	C34	RES	1	-5.065	21.541	9.857	C
HETATM	35	O35	RES	1	-5.568	21.197	8.778	O
HETATM	36	N36	RES	1	-4.812	22.799	10.137	N
HETATM	37	C37	RES	1	-5.263	23.860	9.225	C
HETATM	38	C38	RES	1	3.269	9.738	14.985	C
HETATM	39	C39	RES	1	4.025	8.968	13.906	C
HETATM	40	C40	RES	1	3.827	11.154	15.003	C
HETATM	41	C41	RES	1	3.565	9.074	16.333	C
HETATM	42	H42	RES	1	-4.378	4.032	10.409	H
HETATM	43	H43	RES	1	-4.314	6.146	9.146	H
HETATM	44	H44	RES	1	-2.927	8.024	9.993	H
HETATM	45	H45	RES	1	-1.460	8.870	11.680	H
HETATM	46	H46	RES	1	-1.748	4.653	14.134	H
HETATM	47	H47	RES	1	-0.409	6.483	15.013	H
HETATM	48	H48	RES	1	1.075	11.769	14.731	H
HETATM	49	H49	RES	1	4.961	8.629	14.299	H
HETATM	50	H50	RES	1	3.443	8.126	13.594	H
HETATM	51	H51	RES	1	4.202	9.609	13.068	H
HETATM	52	H52	RES	1	4.491	9.446	16.718	H
HETATM	53	H53	RES	1	2.777	9.297	17.021	H

HETATM	26	N26	RES	1	0.069	8.886	13.943	N
HETATM	27	N27	RES	1	1.377	8.717	14.330	N
HETATM	28	N28	RES	1	-1.552	10.644	13.532	N
HETATM	29	C29	RES	1	-1.795	11.958	13.212	C
HETATM	30	N30	RES	1	-3.127	12.228	12.903	N
HETATM	31	O31	RES	1	-0.941	12.842	13.279	O
HETATM	32	O32	RES	1	-5.405	17.189	11.553	O
HETATM	33	F33	RES	1	-5.696	12.521	12.053	F
HETATM	34	C34	RES	1	-5.041	21.660	9.776	C
HETATM	35	O35	RES	1	-5.377	21.219	8.676	O
HETATM	36	N36	RES	1	-4.845	22.972	10.032	N
HETATM	37	C37	RES	1	-5.036	24.000	9.022	C
HETATM	38	C38	RES	1	3.312	10.179	14.855	C
HETATM	39	C39	RES	1	4.196	9.110	14.180	C
HETATM	40	C40	RES	1	3.756	11.577	14.373	C
HETATM	41	C41	RES	1	3.471	10.091	16.388	C
HETATM	42	H42	RES	1	-3.444	3.042	11.004	H
HETATM	43	H43	RES	1	-2.989	4.633	9.189	H
HETATM	44	H44	RES	1	-1.723	6.783	9.719	H
HETATM	45	H45	RES	1	-0.684	8.223	11.422	H
HETATM	46	H46	RES	1	-1.740	4.775	14.886	H
HETATM	47	H47	RES	1	-0.590	6.917	15.526	H
HETATM	48	H48	RES	1	0.947	11.994	14.283	H
HETATM	49	H49	RES	1	5.246	9.243	14.471	H
HETATM	50	H50	RES	1	3.881	8.102	14.465	H
HETATM	51	H51	RES	1	4.127	9.183	13.090	H
HETATM	52	H52	RES	1	4.518	10.230	16.680	H
HETATM	53	H53	RES	1	2.870	10.865	16.883	H

HETATM	54	H54	RES	1	3.634	8.014	16.201	H	HETATM	54	H54	RES	1	3.142	9.113	16.757	H
HETATM	55	H55	RES	1	4.771	11.172	14.499	H	HETATM	55	H55	RES	1	4.829	11.713	14.557	H
HETATM	56	H56	RES	1	3.145	11.812	14.507	H	HETATM	56	H56	RES	1	3.565	11.696	13.301	H
HETATM	57	H57	RES	1	3.958	11.473	16.016	H	HETATM	57	H57	RES	1	3.225	12.376	14.905	H
HETATM	58	H58	RES	1	-2.310	10.035	13.821	H	HETATM	58	H58	RES	1	-2.287	9.964	13.291	H
HETATM	59	H59	RES	1	-3.819	11.622	13.648	H	HETATM	59	H59	RES	1	-3.730	11.419	12.721	H
HETATM	60	H60	RES	1	-1.749	14.601	12.898	H	HETATM	60	H60	RES	1	-1.875	14.632	13.057	H
HETATM	61	H61	RES	1	-2.754	16.688	12.087	H	HETATM	61	H61	RES	1	-2.860	16.829	12.554	H
HETATM	62	H62	RES	1	-6.592	14.814	12.078	H	HETATM	62	H62	RES	1	-6.562	14.889	11.533	H
HETATM	63	H63	RES	1	-5.604	18.914	9.836	H	HETATM	63	H63	RES	1	-5.530	19.069	9.775	H
HETATM	64	H64	RES	1	-3.826	20.503	14.107	H	HETATM	64	H64	RES	1	-3.836	20.665	14.066	H
HETATM	65	H65	RES	1	-4.419	18.128	13.850	H	HETATM	65	H65	RES	1	-4.294	18.273	13.840	H
HETATM	66	H66	RES	1	-4.313	23.035	10.971	H	HETATM	66	H66	RES	1	-4.466	23.249	10.928	H
HETATM	67	H67	RES	1	-4.540	23.992	8.446	H	HETATM	67	H67	RES	1	-4.301	24.794	9.174	H
HETATM	68	H68	RES	1	-6.203	23.586	8.796	H	HETATM	68	H68	RES	1	-4.915	23.558	8.031	H
HETATM	69	H69	RES	1	-5.372	24.775	9.768	H	HETATM	69	H69	RES	1	-6.034	24.450	9.082	H

Table S2. Optimized ONIOM and docking geometries of ponatinib.

Docking					X	Y	Z		ONIOM					X	Y	Z	
HETATM	1	O4	RES	1	-0.882	13.591	12.949	O	HETATM	1	O4	RES	1	-0.609	13.049	13.173	O
HETATM	2	N7	RES	1	-1.927	11.571	13.544	N	HETATM	2	N7	RES	1	-1.659	11.109	13.741	N
HETATM	3	C24	RES	1	-1.885	12.909	13.129	C	HETATM	3	C24	RES	1	-1.641	12.378	13.230	C
HETATM	4	H56	RES	1	-2.836	11.120	13.612	H	HETATM	4	H56	RES	1	-2.467	10.501	13.537	H
HETATM	5	C22	RES	1	-0.855	10.729	13.839	C	HETATM	5	C22	RES	1	-0.535	10.454	14.263	C
HETATM	6	C20	RES	1	0.286	11.243	14.455	C	HETATM	6	C20	RES	1	0.650	11.106	14.653	C
HETATM	7	C18	RES	1	1.359	10.401	14.750	C	HETATM	7	C18	RES	1	1.749	10.376	15.107	C
HETATM	8	C16	RES	1	1.289	9.046	14.430	C	HETATM	8	C16	RES	1	1.710	8.975	15.198	C
HETATM	9	C19	RES	1	0.148	8.531	13.815	C	HETATM	9	C19	RES	1	0.489	8.353	14.899	C

HETATM	10	C21	RES	1	-0.924	9.374	13.519	C		HETATM	10	C21	RES	1	-0.612	9.063	14.442	C
HETATM	11	C23	RES	1	2.570	10.973	15.411	C		HETATM	11	C23	RES	1	3.026	11.128	15.402	C
HETATM	12	F1	RES	1	2.770	10.508	16.679	F		HETATM	12	F1	RES	1	3.522	10.813	16.630	F
HETATM	13	F2	RES	1	3.731	10.707	14.745	F		HETATM	13	F2	RES	1	4.011	10.837	14.513	F
HETATM	14	F3	RES	1	2.512	12.331	15.522	F		HETATM	14	F3	RES	1	2.870	12.464	15.379	F
HETATM	15	C15	RES	1	2.434	8.116	14.740	C		HETATM	15	C15	RES	1	2.949	8.094	15.262	C
HETATM	16	N5	RES	1	2.211	6.779	14.193	N		HETATM	16	N5	RES	1	3.033	7.422	13.958	N
HETATM	17	C11	RES	1	2.559	5.748	15.178	C		HETATM	17	C11	RES	1	3.905	6.280	13.817	C
HETATM	18	C13	RES	1	2.328	4.345	14.604	C		HETATM	18	C13	RES	1	3.436	5.445	12.612	C
HETATM	19	N6	RES	1	3.097	4.153	13.369	N1+		HETATM	19	N6	RES	1	3.276	6.272	11.362	N
HETATM	20	C14	RES	1	2.752	5.185	12.384	C		HETATM	20	C14	RES	1	2.577	7.595	11.601	C
HETATM	21	C17	RES	1	2.875	2.817	12.821	C		HETATM	21	C17	RES	1	2.598	5.557	10.230	C
HETATM	22	H67	RES	1	4.069	4.240	13.588	H		HETATM	22	H67	RES	1	4.213	6.502	10.991	H
HETATM	23	C12	RES	1	2.982	6.588	12.958	C		HETATM	23	C12	RES	1	3.178	8.293	12.794	C
HETATM	24	C25	RES	1	-3.208	13.552	12.917	C		HETATM	24	C25	RES	1	-2.937	13.041	12.873	C
HETATM	25	C26	RES	1	-3.286	14.922	12.660	C		HETATM	25	C26	RES	1	-2.881	14.439	12.820	C
HETATM	26	C28	RES	1	-4.527	15.524	12.461	C		HETATM	26	C28	RES	1	-4.020	15.203	12.562	C
HETATM	27	C29	RES	1	-5.692	14.759	12.518	C		HETATM	27	C29	RES	1	-5.245	14.544	12.306	C
HETATM	28	C30	RES	1	-5.614	13.390	12.774	C		HETATM	28	C30	RES	1	-5.281	13.153	12.341	C
HETATM	29	C31	RES	1	-7.040	15.371	12.309	C		HETATM	29	C31	RES	1	-6.414	15.366	11.875	C
HETATM	30	C27	RES	1	-4.372	12.787	12.974	C		HETATM	30	C27	RES	1	-4.150	12.390	12.628	C
HETATM	31	C32	RES	1	-4.581	16.931	12.200	C		HETATM	31	C32	RES	1	-4.015	16.631	12.502	C
HETATM	32	C35	RES	1	-4.626	18.112	11.981	C		HETATM	32	C35	RES	1	-4.240	17.824	12.402	C
HETATM	33	C33	RES	1	-4.680	19.516	11.720	C		HETATM	33	C33	RES	1	-4.410	19.215	12.281	C
HETATM	34	N8	RES	1	-4.897	20.109	10.504	N		HETATM	34	N8	RES	1	-4.678	19.885	11.091	N
HETATM	35	N10	RES	1	-5.096	19.457	9.324	N		HETATM	35	N10	RES	1	-4.914	19.329	9.888	N
HETATM	36	C34	RES	1	-4.871	21.477	10.707	C		HETATM	36	C34	RES	1	-4.641	21.256	11.362	C
HETATM	37	C39	RES	1	-5.275	20.262	8.324	C		HETATM	37	C39	RES	1	-5.062	20.186	8.902	C

HETATM	38	C38	RES	1	-5.277	21.743	8.379	C		HETATM	38	C38	RES	1	-5.009	21.601	9.038	C
HETATM	39	C37	RES	1	-5.073	22.303	9.576	C		HETATM	39	C37	RES	1	-4.807	22.146	10.284	C
HETATM	40	N9	RES	1	-4.653	21.768	11.975	N		HETATM	40	N9	RES	1	-4.395	21.465	12.659	N
HETATM	41	C36	RES	1	-4.536	20.556	12.607	C		HETATM	41	C36	RES	1	-4.252	20.234	13.217	C
HETATM	42	H42	RES	1	-5.434	22.336	7.502	H		HETATM	42	H42	RES	1	-5.134	22.229	8.163	H
HETATM	43	H43	RES	1	-5.063	23.368	9.678	H		HETATM	43	H43	RES	1	-4.739	23.213	10.470	H
HETATM	44	H44	RES	1	-5.437	19.814	7.366	H		HETATM	44	H44	RES	1	-5.232	19.722	7.935	H
HETATM	45	H45	RES	1	-4.356	20.436	13.655	H		HETATM	45	H45	RES	1	-4.033	20.120	14.271	H
HETATM	46	H46	RES	1	-6.507	12.802	12.817	H		HETATM	46	H46	RES	1	-6.219	12.659	12.109	H
HETATM	47	H47	RES	1	-4.313	11.737	13.171	H		HETATM	47	H47	RES	1	-4.202	11.304	12.607	H
HETATM	48	H48	RES	1	-2.393	15.510	12.616	H		HETATM	48	H48	RES	1	-1.921	14.913	12.958	H
HETATM	49	H49	RES	1	-1.799	8.980	13.046	H		HETATM	49	H49	RES	1	-1.517	8.549	14.135	H
HETATM	50	H50	RES	1	0.339	12.283	14.701	H		HETATM	50	H50	RES	1	0.741	12.169	14.523	H
HETATM	51	H51	RES	1	0.095	7.491	13.570	H		HETATM	51	H51	RES	1	0.436	7.268	14.938	H
HETATM	52	H52	RES	1	3.333	8.518	14.321	H		HETATM	52	H52	RES	1	3.852	8.661	15.519	H
HETATM	53	H53	RES	1	2.546	8.044	15.802	H		HETATM	53	H53	RES	1	2.843	7.311	16.022	H
HETATM	54	H54	RES	1	4.023	6.715	13.170	H		HETATM	54	H54	RES	1	4.232	8.575	12.607	H
HETATM	55	H55	RES	1	2.678	7.318	12.237	H		HETATM	55	H55	RES	1	2.631	9.225	12.932	H
HETATM	56	H56	RES	1	3.361	5.057	11.513	H		HETATM	56	H56	RES	1	2.677	8.128	10.639	H
HETATM	57	H57	RES	1	1.722	5.081	12.112	H		HETATM	57	H57	RES	1	1.528	7.356	11.797	H
HETATM	58	H58	RES	1	3.589	5.853	15.448	H		HETATM	58	H58	RES	1	4.968	6.554	13.702	H
HETATM	59	H59	RES	1	1.952	5.876	16.050	H		HETATM	59	H59	RES	1	3.835	5.636	14.703	H
HETATM	60	H60	RES	1	2.633	3.615	15.325	H		HETATM	60	H60	RES	1	4.150	4.647	12.387	H
HETATM	61	H61	RES	1	1.287	4.218	14.393	H		HETATM	61	H61	RES	1	2.448	5.024	12.819	H
HETATM	62	H62	RES	1	3.684	2.177	13.104	H		HETATM	62	H62	RES	1	3.202	4.691	9.948	H
HETATM	63	H63	RES	1	1.957	2.422	13.204	H		HETATM	63	H63	RES	1	1.607	5.244	10.565	H
HETATM	64	H64	RES	1	2.821	2.874	11.754	H		HETATM	64	H64	RES	1	2.522	6.274	9.404	H
HETATM	65	H65	RES	1	-6.984	16.099	11.527	H		HETATM	65	H65	RES	1	-6.171	15.750	10.882	H

HETATM	66	H66	RES	1	-7.739	14.609	12.036	H		HETATM	66	H66	RES	1	-7.335	14.790	11.828	H
HETATM	67	H68	RES	1	-7.362	15.843	13.214	H		HETATM	67	H68	RES	1	-6.574	16.240	12.509	H
